# Comparison of Constituents and Antioxidant Activity of Above-Ground and Underground Parts of *Dryopteris crassirhizoma* Nakai Based on HS-SPME-GC-MS and UPLC/Q-TOF-MS

**DOI:** 10.3390/molecules27154991

**Published:** 2022-08-05

**Authors:** Yanjia Wang, Baodong Liu, Xin Wang, Yawen Fan

**Affiliations:** 1 College of Life Science and Technology, Harbin Normal University, Harbin 150025, China; 2 State Key Laboratory of Phytochemistry and Plant Resources in West China, Kunming Institute of Botany, Chinese Academy of Sciences, Kunming 650201, China

**Keywords:** *Dryopteris crassirhizoma*, components, antioxidant activity, total flavonoid content, HS-SPME-GC-MS, UPLC/Q-TOF-MS

## Abstract

*Dryopteris crassirhizoma* Nakai is a Chinese traditional medicinal fern plant for heat-clearing and detoxifying, promoting blood circulation and dissipating blood stasis. Previous researches showed that many factors could influence the components of medicinal plants, and the plant part is one of the main factors. So far, only the underground part of *D. crassirhizoma*, called “Mianma Guanzhong”, has been widely sold in the market. However, the above-ground part was usually at low utilization, resulting in a waste of medicinal resources. In order to further develop and utilize the medicinal resources of *D. crassirhizoma*, the constituents, total flavonoid contents and antioxidant activity of the above-ground and underground parts of *D. crassirhizoma* were tentatively analyzed and compared based on HS-SPME-GC-MS and UPLC/Q-TOF-MS. The results showed that (1) the volatile components were mainly focused in the above-ground part of *D. crassirhizoma*, including 3-carene, isoledene, ionene, 4-amino-1-naphthol and furfural. (2) Nonvolatile components of the underground part of *D. crassirhizoma* contained phenolic acid, flavonoids, phloroglucinol and less fatty acid. (3) The common compounds of the above-ground and underground parts of *D. crassirhizoma* were phenolic acid and flavaspidic acid AB. (4) Antioxidant activity of the underground part was stronger than that of the above-ground part of *D. crassirhizoma*. In conclusion, both the above-ground and underground parts of *D. crassirhizoma* are important medicinal resources worthy of further development.

## 1. Introduction

*Dryopteris crassirhizoma* Nakai is the representative species in Dryopteridaceae [1], distributed in northeastern and northern China, Russia (Far East), Korea and Japan [2]. As a Chinese traditional medicinal fern plant for heat-clearing and detoxifying, promoting blood circulation and dissipating blood stasis [3,4], *D. crassirhizoma* showed many biological functions, such as inhibiting platelet activity [5], antitumor activity [6], antivirus activity [7], etc.

The main compounds in *D. crassirhizoma* were flavonoids [8,9,10], triterpenes [11,12,13], and phloroglucinols [14,15,16]. Previous researches showed that many factors could influence the components of medicinal plants, and the plant part was one of the main factors [17]. For example, ginseng fibrous roots showed high ginsenoside content, and the contents of polyphenolics in different parts of the buckwheat plant were different [17,18]; also, the volatile components in different parts of the flower displayed significant differences [19]. The differences in pharmacological components between the above-ground and underground parts of *Houttuynia cordata* were obvious [20].

As *D. crassirhizoma* is a typical perennial herb, its above-ground parts wither and die in winter and flourish in summer, but the underground part is not severely impacted by the season. So far, the underground part of *D. crassirhizoma*, called “Mianma Guanzhong” (recorded in the Pharmacopoeia of the People’s Republic of China), has been widely sold in the market. However, the above-ground part was usually discarded in the process of collecting medicinal materials, which reduced its utilization rate and resulted in the waste of medicinal resources. Therefore, it is of great significance to study the differences in the constituents and applications of different parts of *D. crassirhizoma*, which could improve the utilization efficiency of its different parts.

Headspace solid-phase micro-extraction gas chromatography-mass spectrometry (HS-SPME-GC-MS) is a new technology for isolating volatiles from plants without solvents and is much more efficient and accurate than the traditional GC method, which tends to lose some compounds and degrade volatiles [20,21]. Ultra-performance liquid chromatography quadrupole time of flight mass spectrometry (UPLC/Q-TOF-MS) has been accepted as one of the common techniques for the analysis of non-volatile ingredients [22].

In order to further develop and utilize the medicinal resources of *D. crassirhizoma*, the constituents of the above-ground and underground parts of *D. crassirhizoma* were tentatively analyzed and compared based on HS-SPME-GC-MS and UPLC/Q-TOF-MS. Additionally, total flavonoid contents and the antioxidant activity of the above-ground and underground parts of *D. crassirhizoma* were determined and compared with the methods of radical scavenging assays, including DPPH, ABTS, etc.

## 2. Results and Discussion

### 2.1. Volatile Components of D. crassirhizoma Determined with HS-SPME-GC-MS 

Volatile components, as vital medicinal resources and essential oils with extensive economic value, are consistently the hotspots of research on angiosperms [17,18,19], but the volatile components of fern are less reported.

**Figure 1:**
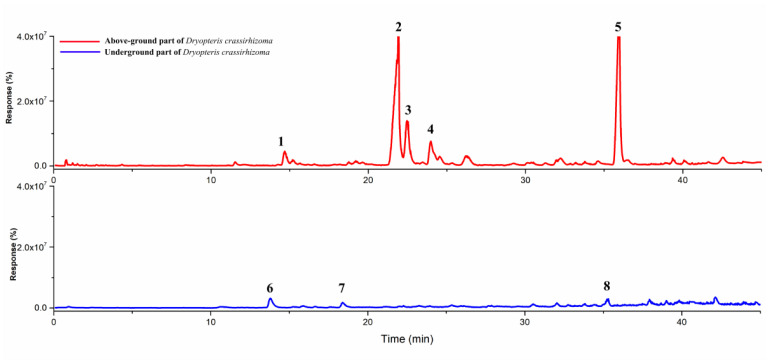
HS-SPME-GC-MS chromatograms of volatile components of above-ground and underground parts from *D. crassirhizoma*.

**Figure 2:**
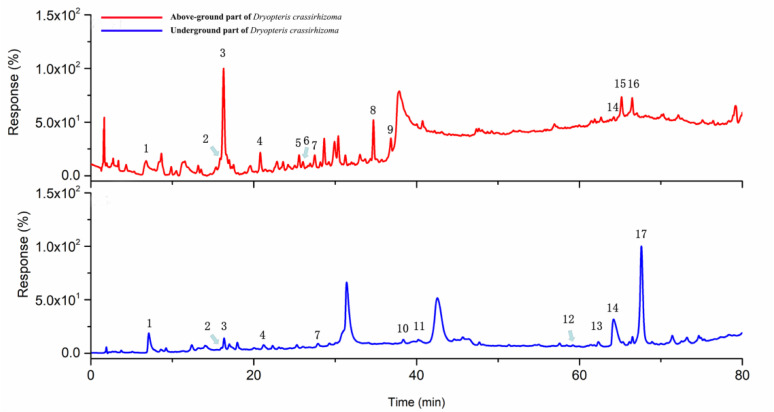
Nonvolatile components of the above-ground and underground of *D. crassirhizoma* determined with UPLC-Q-TOF-MS.

A total of eight volatile components were found in *D. crassirhizoma* with HS-SPME-GC-MS in this paper (Table 1), including furfural, isoledene, 4-amino-1-naphthol, ionene, 3-carene, 3-furaldehyde, 2-acetyl-5-methylfuran and 2-nonadecanol. According to the comparison, it was found that the above-ground and underground components of *D. crassirhizoma* were obviously different (Figure 1). The volatile components were mainly focused in the above-ground part of *D. crassirhizoma,* which mainly contained large proportions of 3-carene (62.50%), isoledene (17.57%), ionene (12.09%), 4-amino-1-naphthol (5.87%) and furfural (1.97%) (Figure 2).

The results showed that the most abundant compound in the above-ground part of *D. crassirhizoma* was 3-carene. As a bicyclic monoterpene, 3-carene is widespread in a variety of plants and shows inhibition activity against multiple microorganisms [23,24]. In addition, as one of the major compounds in pine tree essential oils, 3-carene has anti-inflammatory, antimicrobial, and anxiolytic and sleep-enhancing effects [25]. In recent years, it was reported that 3-carene could delay the growth of bacteria and even lead to cell death [24,26], and is often found in plant essential oils that are used as repellents or show obvious antifungal activity [27,28]. In this paper, it was shown that isoledene is one of the major compounds from the above-ground part of *D. crassirhizoma*, which means the above-ground part of *D. crassirhizoma* could have anti-inflammatory and antimicrobial properties, among others.

Ionene, as one of the aromatic components of green tea and red wines [29], has been identified as a thermal degradation product of β-carotene [30]. Because of its unique structural characteristics, ionene used to be an important application material for various industrial applications [31] and for efficient organic solar cells [32]. In this paper, the proportion of ionene was 12.09%, indicating that as the first-reported compound in fern, ionene deserved further development.

4-Amino-1-naphthol is a water-soluble therapeutic agent that possesses vitamin K activity [33] and can potently inhibit KAT8 (lysine (K) acetyltransferase 8 (KAT8)) [34]. In addition, 4-amino-1-naphthol is a model mediating compound used to stimulate color removal and power production in microbial fuel cells (MFCs) [35].

Furfurals are the most widely distributed simple furan in nature [36], and also one of the most important aromatic compounds in fruits, for example, freeze-dried strawberry [37]. Furfural and its derivatives have been widely applied as fungicides and nematicides, transportation fuels, gasoline additives, lubricants, resins, decolorizing agents, jet fuel blend stocks, drugs, insecticides, bioplastics, flavor enhancers for food and drinks, and rapid all-weather repair systems for bomb-damaged runways and pot holes [36,38,39]. In this paper, 4-Amino-1-naphthol and furfurals were first reported in *D. crassirhizoma,* and their development trend should be paid more attention.

To sum up, it was found the above-ground parts of *D. crassirhizoma* were enriched with volatile constituents possessing important medicinal value and industrial application potential, and deserving of further development.

### 2.2. Nonvolatile Components of D. crassirhizoma Determined with UPLC-Q-TOF-MS 

UPLC/Q-TOF-MS was utilized in the qualitative analysis of nonvolatile components of *D. crassirhizoma*. With reference to the characteristic fragments presented in the spectra and reference spectra available, accurate mass, mass match and isotope pattern match, a total of 14 peaks were tentatively identified (Figure 2). The results showed that nonvolatile components of *D. crassirhizoma* included phenolic acid, flavonoids, phloroglucinols and less fatty acid (Table 2). In terms of their constituents, the above-ground and underground parts of *D. crassirhizoma* were significantly different. The main compounds in the above-ground part were flavonoids, and those in the underground part were phloroglucinols. The common compounds were mostly phenolic acid and flavaspidic acid AB.

With UV_λmax_ at 250 nm, molecular ions at *m*/*z* [M − H]^−^ 463.0882, C_11_H_14_O_6_ was automatically matched by the software, and peak 1 was tentatively identified as marein [40]. Peak 1 with UV_λmax_ at 250 nm and the main ion gave a base peak at *m*/*z* 241.0718, which corresponded to the elenolic acid moiety [40]. Peak 2 with UV_λmax_ at 248, 255 and 283 nm, and molecular ions at *m*/*z* [M − H]^–^ 353.0877, C_16_H_18_O_9_ was automatically matched to and tentatively identified as chlorogenic acid [41]. With the negative ESI-MS spectrum, peak 3 was automatically matched to a molecular ion at *m*/*z* [M − H]^−^ 353.0884, and the characteristic absorption was at 245, 285 and 330 nm. Hence, peak 3 was tentatively identified as neochlorogenic acid [42]. Peak 4 had UV_λmax_ at 245 and 310 nm, *m*/*z* [M − H]^−^ 337.0941, and was tentatively determined as coumaroylquinic acid [29]. Peak 5 had UV_λmax_ at 285 nm, which was similar to marein, molecular ions at *m*/*z* [M − H]^−^ 449.1105, and was automatically matched to C_21_H_22_O_11_ [36]. For peak 7, a molecular ion at *m*/*z* [M − H]^−^ 519.1718 was observed in negative ESI-MS spectrum, the UV spectrum (245 and 280 nm), which indicated this compound might be 6,7 -dihydroxy-2-oxo-1 -benzopyran-4-carboxylic acid [43]. For peak 8, a molecular ion at *m*/*z* [M − H]^−^ 447.0949 was observed in negative ESI-MS spectrum, the UV spectrum (265 nm), which indicated this compound might be flavone, and it was tentatively identified as luteolin-7-o-glucoside [33]. The molecular ions of peak 12 were at *m*/*z* [M − H]^−^ 389.1246 in the negative ESI-MS spectrum, and automatically matched to C_20_H_22_O_8_; with the UV_λmax_ at 283 nm, it was tentatively identified as 7-hydroxyl-4, 3, 5, 6, 8-pentamethoxyflavone [44]. Base on the characteristic fragments of accurate mass, mass match and isotope pattern match and reference spectra available, peaks 13, 14 and 17 were tentatively identified as flavaspidic acid AP, flavaspidic acid AB and flavaspidic acid PB, respectively [4].

Phenolic acids have recently gained substantial attention due to their various practical, biological and pharmacological effects [45]. It was found that *Dryopteris crassirhizoma* is rich in phenolic acids, such as common elenolic acid, chlorogenic acid, neochlorogenic acid and coumaroylquinic acid. Chlorogenic acid and neochlorogenic acid are the most important and are widely used medicinal ingredients at present. Chlorogenic acid shows higher antioxidant activity, antibacterial, anti-inflammatory, antipyretic, anti-obesity, antiviral, anti-microbial and anti-hypertension properties, and could be used as a hepatoprotective, cardioprotective, and neuroprotective agent, a central nervous system (CNS) stimulator, and modulator of lipid metabolism and glucose [45]. In addition, it was reported that neochlorogenic acid could be a suppressive component of the LPS-induced inflammatory response in A549 cells [46], inhibit lipopolysaccharide-induced activation and pro-inflammatory responses in BV2 microglial cells, and effectively trigger robust MCU-mediated calcium overload in cancer therapy [47,48].

The underground part of *D. crassirhizoma*, called “MianMa GuanZhong”, is a famous traditional Chinese medicinal material, recorded in the Pharmacopoeia of the People’s Republic of China. Although, it was proved that flavonoids and phloroglucinols are its representative compounds [13,14,15,16], the contents of phloroglucinols and flavonoids are lower than those of phenolic acids and fatty acids. According to the results of this paper, the phloroglucinols detected in 1 g samples (from the underground part of *D. crassirhizoma*) via UPLC/Q-TOF-MS were flavaspidic acid AB, flavaspidic acid AP and flavaspidic acid PB, which are the same as medicinal components in the drug Mianma Guanzhong.

**Table 2:** Qualitative analysis of nonvolatile components of *D. crassirhizoma* by UPLC/Q-TOF-MS.

No.	RT (Min)	Compound Formula	Molar Mass	Experimental [M − H]^−^ *m*/*z*	ppm	UV (nm) λmax	Identification	Type	Part	Ref.
1	6.884	C_11_H_14_O_6_	242.0791	241.0718	−0.19	250	Elenolic acid	Phenolic acid	AB	[40]
2	16.111	C_16_H_18_O_9_	354.0954	353.0877	−0.9	248,255,283	Chlorogenic acid	Phenolic acid	AB	[41]
3	16.795	C_16_H_18_O_9_	354.0954	353.0884	−1.53	245,285,330	Neochlorogenic acid	Phenolic acid	AB	[42]
4	20.9	C_16_H_18_O_8_	338.1014	337.0941	−3.68	245,310	Coumaroylquinic acid	Phenolic acid	AB	[29]
5	25.657	C_21_H_22_O_11_	450.1178	449.1105	−3.45	285	Marein	Flavonoids	A	[36]
6	26.5	C_14_H_24_O_8_	320.1481	319.1408	−2.92	258	Unknown	_	A	—
7	27.524	C_22_H_32_O_14_	520.179	519.1718	0.31	245,280	6,7-Dihydroxy-2-oxo-1-benzopyran-4-carboxylic acid	Phenolic acid	AB	[33]
8	34.8	C_21_H_20_O_11_	448.1022	447.0949	−3.56	265	Luteolin-7-O-glucoside	Flavonoids	A	[33]
9	37	C_11_H_12_O_4_	208.0743	207.0670	−3.33	245	Unknown	_	A	—
10	38.152	C_10_H_12_O_4_	196.0734	195.0661	0.93	248,280	Methyl veratrate	Phenolic acid	B	[49]
11	40.021	C_10_H_12_O_4_	196.0736	195.0663	−0.2	248,280	Hydroxyconiferyl alcohol	Phenolic acid	B	[50]
12	59.042	C_20_H_22_O_8_	390.1319	389.1246	−1.03	283	7-Hydroxyl-4,3,5,6,8-pentamethoxyflavone	Flavonoids	B	[44]
13	62.146	C_21_H_24_O_8_	404.1479	403.1406	–1.91	290	Flavaspidic acid AP	Phloroglucinol	B	[4]
14	65.032	C_22_H_26_O_8_	418.1635	417.1562	0	245	Flavaspidic acid AB	Phloroglucinol	AB	[4]
15	65.8	C_12_H_26_O_8_	398.1619	397.1546	2.99	250	Unknown	_	A	—
16	66.55	C_18_H_32_O_3_	296.2366	295.2293	−4.91	245	Hydroxyoctadecadienoic acid	Fatty acid	A	[35]
17	67.819	C_23_H_28_O_8_	432.1795	431.1723	−2.6	245,295	Flavaspidic acid PB	Phloroglucinol	B	[4]

A. above-ground part of *Dryopteris crassirhizoma*; B. underground part of *Dryopteris crassirhizoma*.

### 2.3. Comparison of Total Flavonoid Content and Antioxidant Activity of Above-Ground and Underground Parts of Dryopteris crassirhizoma 

**Figure 3:**
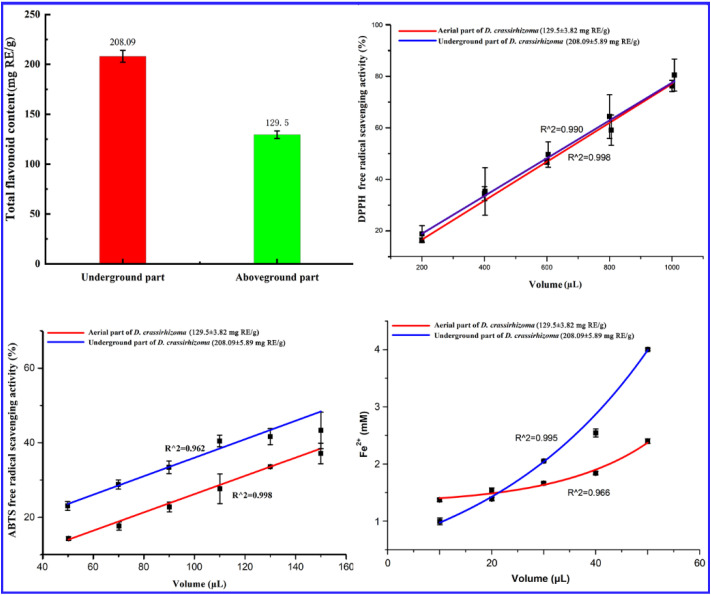
Comparison of total flavonoid content and antioxidant activities of above-ground and underground parts of *Dryopteris crassirhizoma*.

The total flavonoid content in the above-ground and underground parts of *D. crassirhizoma* was determined to be 129.5 ± 3.82 mg RE/g and 208.09 ± 5.89 mg RE/g, respectively. The total flavonoid content in the underground part was two times higher than that in the above-ground part (Figure 3), which was higher than that in most species of *Lindsaeaceae* and *Blechnaceae* [51], and also higher than the total flavonoid content in bryophytes [52].

The potent free radical-scavenging and anti-oxidative activity of this medicinal plant might result from its high contents of flavonoid-type compounds [51]. With increasing volume, the DPPH free radical scavenging potential of *D. crassirhizoma* observably increased (Figure 3). Roughly 1 mL of extract from the underground and above-ground parts could scavenge about 80% and 76% of free radicals, respectively. With increasing volume, the ABTS free radical scavenging potential also observably increased (Figure 3). The ABTS free radical scavenging potential of the underground part was higher than that of the above-ground part. Within the range of 20 μL to 50 μL, the Fe^3+^ reducing capacity of the underground part of *D. crassirhizoma* was higher than that of the above-ground part (Figure 3).

Based on the above results, it was shown that the antioxidant activities of the underground part were stronger than those of the above-ground part of *D. crassirhizoma*, and the antioxidant activities of *D. crassirhizoma* also were obviously stronger than those of most reported ferns and bryophytes, especially in terms of ABTS-free radical scavenging activities [53].

## 3. Materials and Methods

### 3.1. Plant Materials

**Figure 4:**
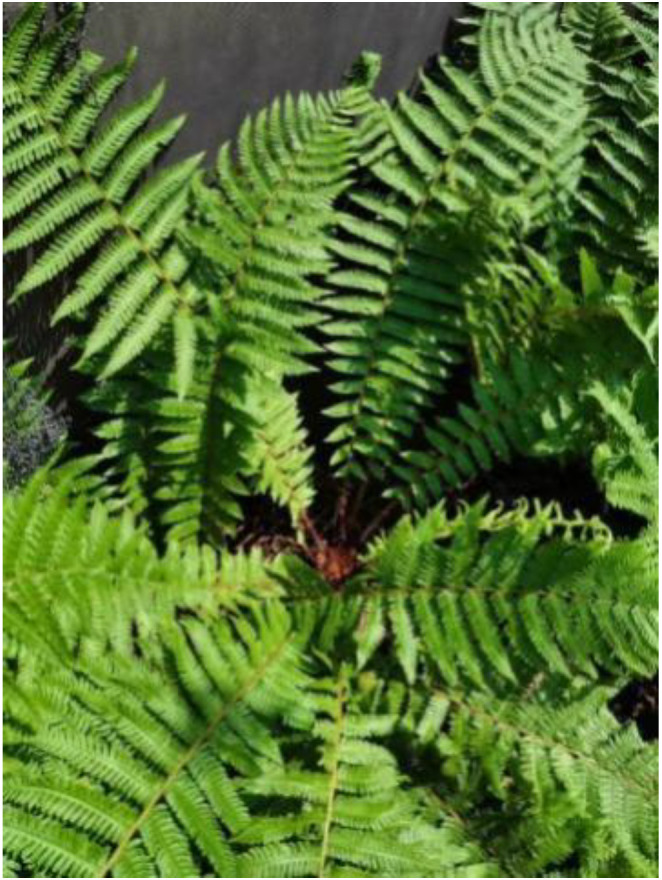
*D. crassirhizoma*.

*D.crassirhizoma* (Figure 4) was collected on 29 September 2020 from the Botanical Garden of Harbin Normal University, where a greenhouse served for scientific research and teaching with proper temperature (18–35 °C), luminous intensity (1500 Lx–3000 Lx) and relative humidity (35–80%).

### 3.2. Chemicals and Reagents 

Rutin (purity > 99.0%), 2,2-diphenyl-1-picrylhydrazyl (DPPH), 2,2-azinobis-(3-ethyl -benzothiazoline-6-sulfonic acid) (ABTS), and 2,4,6-tri-2-pyridyl-s-triazine (TPTZ) were purchased from Sigma Co. (Shanghai, China). Methanol was HPLC grade. All other reagents and solvents (FeCl_3_, FeSO_4_) were of analytical grade. All aqueous solutions were prepared using newly double-distilled water.

### 3.3. Preparation of Plant Extracts 

HS-SPME-GC-MS Analysis:

One gram each of fresh and cleaned above-ground part and underground part of *D. crassirhizoma* were prepared for HS-SPME-GC-MS analysis.

UPLC-Q-TOF-MS Analysis:

Fresh *D. crassirhizoma* was divided into above-ground and underground parts, and then cleaned by double-distilled water and dried in outdoor air. The treatment was similar with the processes for materials from Mianma Guanzhong. After drying in the shade, samples were dried at 75 °C for 48 h in an electro-thermostatic blast oven (Bluepard Instruments CO., LTD, Shanghai, China). Finally, materials were powdered by a pulverizer and filtered through 40-mesh screen.

One gram samples were taken from the above-ground part and underground part, respectively, and individually extracted with 25 mL of 70% ethanol for 2 h at 50 °C in a thermostat water bath. The mixture was extracted with an ultrasound apparatus for 20 min, and the extract collected via a vacuum suction filter pump. The above operation was repeated once more, and the extract collected a second time. The mixture was prepared for the UPLC-Q-TOF-MS analysis and the determination of total flavonoids content and antioxidant activity.

### 3.4. Headspace Solid-Phase Microextraction and Gas Chromatography-Mass Spectrometry (HS-SPME-GC-MS) Analysis 

The Aglient 7890A gas chromatograph coupled to a 5975C mass-selective detector was used for the analysis. The PDS (100 µm) SPME fiber was selected, which showed a superior extraction capability for the various volatiles. The SPME fiber was desorbed for 1 min in splitless mode. The flow rate of the helium carrier gas was 0.72 mL/min. Analytes were separated on an HP-5MS capillary column (3.0 m × 0.25 mm, 0.25 mm, Agilent Technologies, Santa Clara, CA, USA). The GC oven temperature program was set at an initial temperature of 50 °C for 1 min, raised to 120 °C at 3 °C/min, held for 2 min, raised to 220 °C at 4 °C/min, then held for 60 min. The MSD was operated with electron impact ionization in selected ion monitoring (SIM) mode. The list of ions selected for each analyte is summarized in Table 1. The GC-MS interface and MS system source temperatures were 280 and 250 °C, respectively.

### 3.5. Ultra-Performance Liquid Chromatography Quadrupole Time of Flight Mass Spectrometry (UPLC-Q-TOF-MS) Analysis 

The Agilent 1290 UPLC (Agilent Technologies, Santa Clara, CA, USA) was used to perform the chromatographic separation. The UV spectrum was recorded between 190–380 nm; the UV detector was set at 254 nm. An Agilent ZORBAX SB-C18 (4.6 × 150 mm; i.d. 5.0 mm) column using a gradient elution (methanol (A)/water (0.5% HCOOH) (B)) was chosen. All of the MS experiments were conducted on an Agilent 6540 Q-TOF-MS equipped with electrospray ionization (ESI) interface (Agilent Technologies, USA).

The gradient condition was 0–80 min at 5–100% A. The column temperature was set at 25 °C, the flow rate was kept at 1 mL/min, and the injection volume was 2 μL. The MS analysis was performed in negative scan modes under the following operation parameters: the voltage was set at 160 V. Full scan data acquisition and dependent scan event data acquisition were performed from *m*/*z* 100–1200.

### 3.6. Determination of Total Flavonoids Content and Antioxidant Activity 

#### 3.6.1. Determination of Total Flavonoids Content

The method of determination of total flavonoids content was a colorimetric assay (NaNO_2_–Al (NO_3_)_3_–NaOH), which was the same as in a previous report [54].

#### 3.6.2. DPPH Assay

The method of DPPH scavenging activity was same as our previous report [51]. Briefly, a solution of DPPH (0.1 mM) in ethanol mixed with different concentrations of extract (1.0 mL) was incubated for 30 min, and then the absorbance value at 517 nm was recorded. Ethanol (70%) was used as the control group. The DPPH scavenging activity was calculated using the following formula:

DPPH free radical scavenging activity (%) = (1 − A_sample 517_/A_control 517_) × 100 (1)

All of the experiments were performed in triplicate (*n* = 3) and found to be reproducible within the margins of experimental error (RSD < 5.0%).

#### 3.6.3. ABTS Assay

The ABTS free radical scavenging activity was measured with colorimetry [55]. First, a mixture of ABTS and potassium persulfate was stored in the dark for 16 h before use. One-hundred fifty microliter volumes of extracts in different concentrations were added to fittingly diluted ABTS solutions. The absorbance at 734 nm was recorded for 6 min. In the control, ethanol was substituted for the sample. ABTS free radical scavenging activity was determined with following formula:

ABTS free radical scavenging activity (%) = (1 − A_sample 734_/A_control 734_) × 100. (2)

All of the experiments were performed in triplicate (*n* = 3) and found to be reproducible within the margins of experimental error (RSD < 5.0%).

#### 3.6.4. FRAP Assay

The FRAP assay was carried out according to our previous report [54]. The FRAP reagent (10 mM TPTZ in 40 mM HCl solution and 20 mM FeCl_3_ in 0.25 L acetate buffer (pH 3.6)) was used immediately after preparation. Fifty microliter extracts in variable concentrations and 1.5 mL FRAP reagent were mixed for 4 min. The absorbance at 593 nm was recorded. Calibration curves for FeSO_4_ were used to determine the results. All of the experiments were performed in triplicate (*n* = 3) and found to be reproducible within the margins of experimental error (RSD < 5.0%).

## 4. Conclusions

The volatile components were mainly focused in the above-ground part of *D. crassirhizoma*, which shows important medicinal value and industrial application potential. Phenolic acid, flavonoids, phloroglucinol and less fatty acid were the main compounds in the underground part of *D. crassirhizoma*. Additionally, total flavonoid content and antioxidant activity in the underground part were higher and stronger, respectively, than those in the above-ground part of *D. crassirhizoma*. Therefore, the above-ground and the underground parts of *D. crassirhizoma* are important medicinal resources worthy of further development.

## Figures and Tables

**Table 1:** Volatile components of *D. crassirhizoma determined* *with HS-SPME-GC-MS*.

No.	RT (min)	Volatile Component	Molecular Formula	Proportion (%)	PW 50% (min)
1	14.649	Furfural	C_5_H_4_O_2_	1.97 (AP)	0.243
2	21.65	Isoledene	C_15_H_24_	17.57 (AP)	0.381
3	22.44	4-Amino-1-naphthol	C_10_H_9_NO	5.87 (AP)	0.19
4	23.978	Ionene	C_13_H_18_	12.09 (AP)	0.638
5	35.94	3-Carene	C_10_H_16_	62.50 (AP)	0.695
6	13.783	3-Furaldehyde	C_5_H_4_O_2_	22.04 (UP)	0.243
7	18.316	2-Acetyl-5-methylfuran	C_7_H_8_O_2_	3.03 (UP)	0.11
8	35.888	2-Nonadecanol	C_19_H_40_O	1.01 (UP)	0.09

AP, above-ground part of *Dryopteris crassirhizoma*; UP, underground part of *Dryopteris crassirhizoma*; PW, peak width.

## Data Availability

Data are available from the corresponding author on request.
